# Identification and antimicrobial susceptibility testing of Gram-positive and Gram-negative bacteria from positive blood cultures using the Accelerate Pheno™ system

**DOI:** 10.1007/s10096-019-03703-y

**Published:** 2019-10-22

**Authors:** Måns Ullberg, Volkan Özenci

**Affiliations:** 1 Division of Clinical Microbiology, Department of Laboratory Medicine, Karolinska Institutet, Stockholm, Sweden; 2 Department of Clinical Microbiology, Karolinska University Hospital, Huddinge, SE 141 86 Stockholm, Sweden

## Abstract

Rapid identification and antimicrobial susceptibility testing remain a crucial step for early efficient therapy of bloodstream infections. Traditional methods require turnaround times of at least 2 days, while rapid procedures are often associated with extended hands-on time. The Accelerate Pheno™ System provides microbial identification results within 90 min and susceptibility data in approximately 7 h directly from positive blood cultures with only few minutes of hands-on time. The aim of this study was, therefore, to evaluate the performance of the Accelerate Pheno™ System in identification and antimicrobial susceptibility testing of both Gram-positive and Gram-negative bacteria directly from clinical blood culture samples. We analyzed 108 and 67 blood culture bottles using the Accelerate PhenoTest™ BC kit with software version v1.0 and the FDA-cleared version v1.2, respectively. Reliable identification was achieved for Enterobacteriaceae, staphylococci, and enterococci, with 76/80 (95%), 42/46 (91%), and 10/11 (91%) correct identifications. Limitations were observed in the identification of streptococci, including *Streptococcus pneumoniae* and *Streptococcus pyogenes*, and coagulase-negative staphylococci. Antimicrobial susceptibility results for Enterobacteriaceae, for amikacin, ertapenem, ciprofloxacin, gentamicin, meropenem, and piperacillin-tazobactam ranged between 86 and 100% categorical agreement. Using v1.2, results for ceftazidime showed 100% concordance with the reference method. For staphylococci, the overall performance reached 92% using v1.2. Qualitative tests for detection of methicillin or macrolide-lincosamide-streptogramin B (MLSB) resistance caused major and very major errors for isolates. Overall, the present data show that the Accelerate Pheno™ system can, in combination with Gram stain, be used as a rapid complementation to standard microbial diagnosis of bloodstream infections.

## Introduction

Bloodstream infections are associated with high morbidity and mortality and remain a leading cause of death [3]. Early effective therapy improves the disease outcomes but is dependent on identification and susceptibility testing of the causative agent [2].

Automated antimicrobial susceptibility testing (AST) systems such as the Vitek 2 system (bioMeriéux) and Phoenix 100 (BD Biosciences) use simplified protocols and require shorter incubation times compared to standard methods. Also, these procedures can be combined with rapid protocols to considerably reduce the time until a resistance profile is available [14, 20, 23]. These rapid procedures are, however, associated with extended hands-on time and the need for specialized personnel and are, thus, not compatible with the implementation of automated methods.

Time-lapse microscopic imaging of bacterial colony formation in the presence of an antimicrobial agent has been suggested as a novel approach to ascertain AST directly from the positive blood culture bottles [8]. The Accelerate Pheno™ system (Accelerate Diagnostics Inc., Tucson, AZ, USA) employs this technique in a fully automated system. The system identifies 15 bacterial species/groups as well as two *Candida* species and determines phenotypic AST for part of the identification panel. Bacteria or yeast cells are identified by fluorescence in situ hybridization (FISH). In addition to specific probes, bacterial and eukaryotic cells are universally labeled which allows to detect the presence of off-panel microorganisms. Microorganisms are then subjected to species-specific AST. Morphokinetic cellular parameters, such as division rate and cell morphology, are recorded and a minimal inhibitory concentration (MIC) for each antibiotic is calculated using the software algorithm. Results are automatically reported after approximately 90 min for identification and 7 h for antimicrobial susceptibility.

Only a few studies that examined the clinical performance of the Accelerate PhenoTest™ BC are available. The published studies focused on a limited group of microorganisms or patients, Gram-negative bacteria or pediatric oncology patients, respectively [7, 18]. Moreover, the FDA-cleared Accelerate Pheno™ system employs a different software version than the version used in those studies. The aim of the current study was, therefore, to evaluate the performance of the Accelerate Pheno™ system, including the FDA-cleared version of the software, on a broader selection of clinical blood culture samples including Gram-positive and Gram-negative bacteria.

## Materials and methods

### Blood culture bottles

A total of 175 positive blood culture bottles were chosen from clinical blood culture samples sent to Karolinska University Laboratory, Stockholm, Sweden, from three tertiary care hospitals in the greater Stockholm area. Blood culture bottles BacT/Alert FA Plus, BacT/Alert FN Plus, and BacT/Alert PF Plus were incubated in the BacT/Alert 3D system. Bottles were selected per the following criteria: (i) ≤ 8 h after blood culture positivity, (ii) growth in aerobic bottles, (iii) time to positivity < 24 h, and (iv) positivity in more than one bottles from the same patient. Referring to the manufacturer’s protocol, the first point (i) was required per manufacturer’s specifications while samples that did not meet the remaining criteria were investigated only in cases where no other bottles were available such as Gram stain results and patient data, as available from the laboratory information system, were considered in cases where more than two suitable positive blood culture bottles were available. Samples suspected for contamination or off-panel organisms were avoided. Comparisons of results were made using samples taken from the same blood culture bottle.

### Standard procedures for identification and susceptibility testing

Microorganisms from positive blood culture bottles were initially assessed using Gram stain. Species identification was determined by MALDI-TOF MS (Bruker Daltonik, Bremen, Germany) in nearly all cases. In the event of failure of identification by MALDI-TOF MS, Vitek 2 was used. Antimicrobial susceptibility for all isolates included in this study was tested by disk diffusion following EUCAST guidelines and breakpoints according to version 6.0. In addition, Enterobacteriaceae were tested with the Vitek 2 GN AST-N218 card. MIC values were interpreted following EUCAST breakpoints as above.

### ID and AST with the Accelerate Pheno™ system

The Accelerate PhenoTest™ BC kit (Accelerate Diagnostics, Inc., Tucson, AZ, USA) was run according to manufacturer’s instructions. In brief, an aliquot of 2–5 ml blood culture broth was aseptically removed from the blood culture bottle and transferred to a sample vial and, regardless of inoculation volume, a 0.3-ml aliquot was processed and analyzed by the system. The run was then immediately started. After completing the run, the blood culture broth from the sample vial was cultured on blood agar plates and suitable selective agar plates, if indicated. All isolated bacteria were stored at − 80 °C. The first set of samples (*n* = 108, collected between April and September 2016) was run and evaluated by the Accelerate host software version v1.0.0.417 (v1.0); the second set of samples (*n* = 67, collected between January and March 2017) was run and evaluated by the Accelerate host software version v1.2.0.87 (v1.2). Quality control runs were performed each week the instrument was in use, alternating between the two modules available during the study period.

### Data evaluation and statistics

AST results were analyzed for categorical agreement (S, susceptible; I, intermediate; R, resistant). Categorical errors were defined as minor (S/R for I, or I for S/R by reference method), major (R for S by reference method, false resistant), or very major (S for R by reference method, false sensitive) errors.

All data were evaluated using GraphPad Prism 6.00, GraphPad Software, La Jolla, CA, USA. Continuous data are presented with median and range, and results were compared using a Mann-Whitney test. Categorical data are given in absolute numbers and percentages, results were compared using Fisher’s exact test. Differences with *P* values < 0.05 were considered statistically significant.

## Results

### Samples and technical performance

**Fig. 1:**
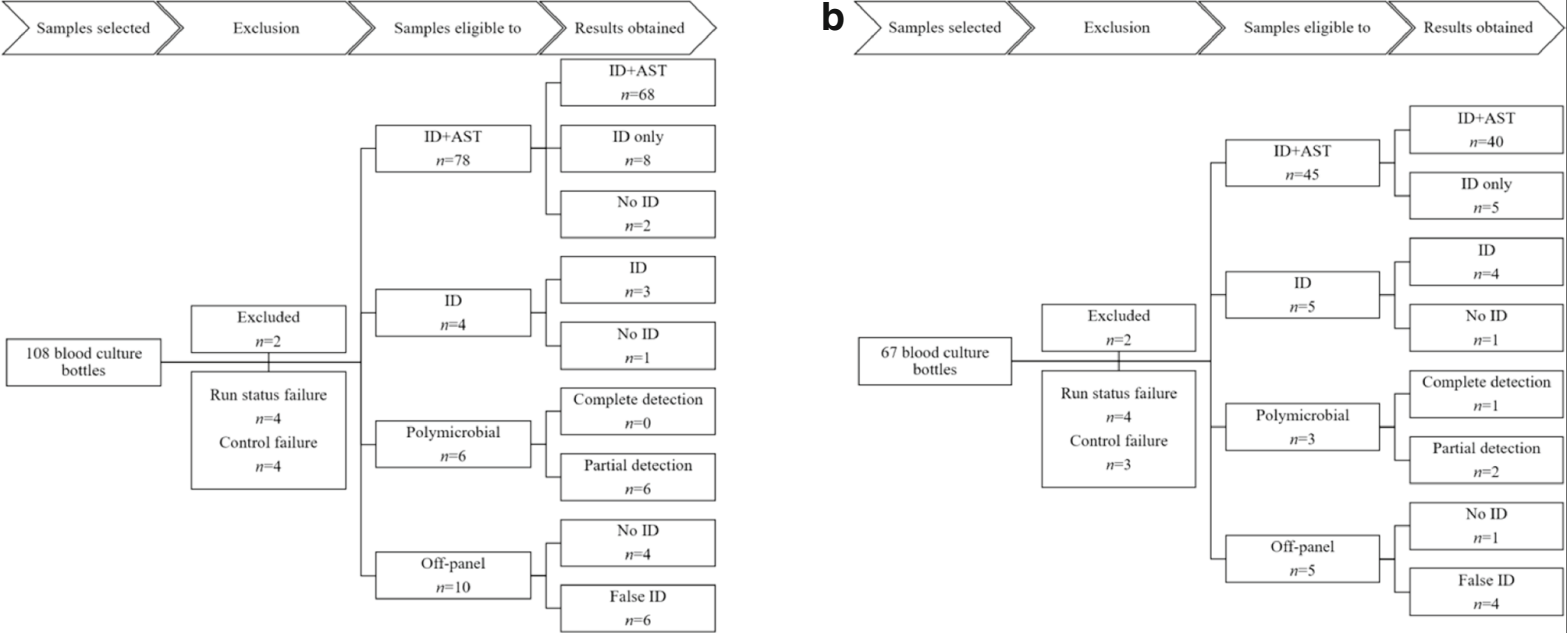
Blood culture samples included in the study. Positive blood culture bottles were analyzed with the Accelerate PhenoTest™ BC using software version SW1.0 (**a**) or SW1.2 (**b**). ID, identification; AST, antimicrobial susceptibility testing

Blood culture bottles were selected based on the manufacturer’s instructions and microbiological parameters including time to detection, aerobic growth, and the number of positive bottles from the same patient. These criteria were chosen to focus on clinically relevant samples. During the first study period, a total of 108 bottles were selected and analyzed using the Accelerate host software version v1.0 (Fig. 1a). In the second study period using the new or software version, a total of 67 bottles was evaluated and software version v1.2 was applied (Fig. 1b). An overview of pre-analytical sample parameters and technical assay performance is shown in Table 1. From the initially chosen samples, ten and nine bottles from the first and second study periods, respectively, were excluded from evaluation. In the majority of cases, run status failure was related to cassette illumination failure and the run was terminated after 6–8 min. In one case, run status failure was reported at the end of the run at 8.25 h. Control failures were reported at the end of FISH analysis and bacterial identification failures were reported at 82 min. These failures gave an error message “Too few cells for analysis” in the Accelerate run report. Cultures from the blood culture broth did however not indicate low bacterial counts or impaired bacterial viability; nor was there a delay in starting the assay after blood culture positivity (3.4 h for failed samples versus 4.5 h for successfully analyzed samples, *P* = 0.42). In contrast, the time to detection in the blood culture system was significantly longer for samples with control failure (21 versus 14.5 h, *P* = 0.005). In each part of the study, two samples were excluded for reasons unrelated to the system, i.e., failures in the control culture, duplicate samples, or electricity breaks during the analysis. With these failures excluded, 15/171 (8.8%) runs could not be analyzed because of failures in the system.

**Table 1:** Sample characteristics and technical performance of the Accelerate PhenoTest™ BC kit

	v1.0	v1.2	*P* value
Pre-analytical sample characteristics			
Time to detection [h]^a^	14 (5–27)	15 (6–54)	0.1692
Time until analysis [h]^a^	4.5 (0.6–15.6)	4.1 (0.3–7.6)	0.0436
Assay performance			
Run status failure^b^	4/106 (3.8%)	4/65 (6%)	0.4807
Control failure^b^	4/102 (3.9%)	3/61 (5%)	1.000
Time to ID [min]^a^	82 (80–84)	81 (80–85)	<0.0001
Time to AST [h]^a^	6.7 (6.5–6.9)	6.5 (6.5–6.9)	0.0002
Completed ID + AST results (eligible samples)	68/78 (87%)	40/45 (89%)	0.7807

^a^Data are presented as median (range) and evaluated by Mann-Whitney test

^b^Data are presented as *n* (%) and evaluated by Fisher’s exact test

Results reporting bacterial identification and AST obtained with v1.0 and v1.2 are presented separately in “Performance using version v1.0” and “Evaluation of v1.2” sections, respectively.

### Performance using software version v1.0

#### Bacterial identification

Samples from the first study period contained 105 isolates and 94/105 (89.5%) were covered by the PhenoTest™ BC kit panel. Samples containing species that were not differentiated by the panel were counted as a single isolate, e.g., coagulase-negative staphylococci (CoNS) or α-hemolytic streptococci. As determined by Gram staining, the 105 isolates included 59 Gram-negative rods, 26 staphylococci, 11 streptococci, and 9 enterococci (Table 2). The performance of the PhenoTest™ BC kit for bacterial identification is summarized in Table 3. Identification of Gram-negative rods, staphylococci, and enterococci was highly reliable, with 84/94 (89%) of bacteria detected and correctly identified. Undetected organisms were primarily found in polymicrobial infections. In contrast, cross-reactivity was observed among the group of α-hemolytic streptococci, including three α-hemolytic streptococci reported as *S. pneumoniae*. Two of three off-panel β-hemolytic *Streptococcus* species were reported as CoNS. In addition, three *Candida* species and one of each *Escherichia coli*, *Pseudomonas aeruginosa*, and *Streptococcus* species were reported by the PhenoTest™ BC kit while not observed using standard culture. Another 13 isolates were reported as “indeterminate” (not depicted in Table 2), including *Enterobacter* species (*n* = 5, all in combination with *Klebsiella* or *Citrobacter* species), *Staphylococcus* species (*n* = 7), and *Enterococcus faecium* (*n* = 1). None of these isolates could be detected by standard methods, nor were there any other microbiological results from these patients indicating the presence of such microorganisms. Thus, in total 19 detections were regarded false positive.

**Table 2:** Identification results from Accelerate PhenoTest™ BC kit in comparison to standard methods

Gram stain	Samples (*n*)	Standard culture	PhenoTest™ BC kit
SW1.0			
Gram-negative rods	26	*Escherichia coli*	*Escherichia coli*
	1	*Escherichia coli*, ***Pseudomonas aeruginosa***	*Escherichia coli*, ***Candida albicans***
	14	*Klebsiella* species	*Klebsiella* species
	1	*Klebsiella pneumoniae*	*Klebsiella* species, ***Candida albicans***
	1	*Klebsiella oxytoca*	*Klebsiella* species, ***Streptococcus*species**
	1	*Klebsiella oxytoca*	*Klebsiella* species, ***Escherichia coli***
	1	***Klebsiella oxytoca***	**Unidentified organism(s)**
	3	*Enterobacter* species	*Enterobacter* species
	1	*Enterobacter cloacae*, ***Escherichia coli***^a^	*Enterobacter* species, **unidentified organism(s)**
	1	***Enterobacter cloacae***	**Unidentified organism(s)**
	2	*Citrobacter* species	*Citrobacter* species
	1	*Proteus mirabilis*	*Proteus* species
	1	*Haemophilus influenzae*	Unidentified organism(s)
Gram-negative rods, Gram-positive cocci in chains/diplococci	1	*Enterobacter cloacae*, ***Enterococcus faecium***	*Enterobacter* species
	1	*Escherichia coli*, ***Klebsiella pneumoniae*,*Enterococcus durans***^b^	*Escherichia coli*
Gram-positive cocci in clusters	12	*Staphylococcus aureus*	*Staphylococcus aureus*
	1	*Staphylococcus aureus*	*Staphylococcus aureus*, ***Pseudomonas aeruginosa***
	1	*Staphylococcus aureus*, ***Staphylococcus epidermidis***	*Staphylococcus aureus*
	8	Coagulase-negative staphylococci	Coagulase-negative staphylococci
	1	*Staphylococcus warneri*	Coagulase-negative staphylococci
	1	*Staphylococcus simulans*	Unidentified organism(s)
Gram-positive cocci in clusters, Gram-positive cocci in chains/diplococci	1	*Enterococcus faecalis*, ***Staphylococcus aureus***	*Enterococcus faecalis*
Gram-positive cocci in chains/diplococci	5	*Enterococcus faecalis*	*Enterococcus faecalis*
	1	*Enterococcus faecium*	*Enterococcus faecium*
	1	*Streptococcus agalactiae*	*Streptococcus agalactiae*
	1	*Streptococcus pneumoniae*	*Streptococcus pneumoniae*
	1	***Streptococcus mitis*-group**	**Unidentified organism(s)**
	1	***Streptococcus mitis*-group**	***Streptococcus pneumoniae***
	1	***Streptococcus pyogenes***	**Coagulase-negative staphylococcus**
	1	***Streptococcus dysgalactiae***	**Coagulase-negative staphylococcus**
	2	*Streptococcus dysgalactiae*	Unidentified organism(s)
	2	***Streptococcus sanguinis*-group**	***Streptococcus pneumoniae***
	1	*Streptococcus salivarius*-group	*Streptococcus* species, ***Candida glabrata***
SW1.2			
Gram-negative rods	14	*Escherichia coli*	*Escherichia coli*
	5	*Klebsiella* species	*Klebsiella* species
	3	*Enterobacter* species	*Enterobacter* species
	1	***Stenotrophomonas maltophilia***	**Coagulase-negative staphylococci**
	1	*Streptococcus anginosus*, ***Haemophilus influenzae***	*Streptococcus* species, **coagulase-negative staphylococci**
Gram-positive cocci in clusters	11	*Staphylococcus aureus*	*Staphylococcus aureus*
	2	***Staphylococcus aureus***	**Coagulase-negative staphylococci**
	7	Coagulase-negative staphylococci	Coagulase-negative staphylococci
	1	Coagulase-negative staphylococci, ***Streptococcus salivarius*-group**	Coagulase-negative staphylococci
Gram-positive cocci in clusters, Gram-positive cocci in chains/diplococci	1	*Enterococcus faecalis*	*Enterococcus faecalis*
Gram-positive cocci in chains/diplococci	1	*Enterococcus faecium*	*Enterococcus faecium*
	1	*Enterococcus faecium*, *Staphylococcus epidermidis*, *S. haemolyticus*	*Enterococcus faecium*, coagulase-negative staphylococci
	2	*Streptococcus mitis/oralis*	*Streptococcus* species
	1	*Streptococcus agalactiae*	*Streptococcus agalactiae*
	1	***Streptococcus pneumoniae***	***Streptococcus agalactiae***
	1	***Streptococcus pneumoniae***	**Four or more organisms detected.**
	1	*Streptococcus pyogenes*	*Streptococcus* species
	1	*Streptococcus gordonii*	*Streptococcus* species
	1	*Abiotrophia defectiva*	Suspected off-panel microorganism
Gram-positive rods	1	***Arcanobacterium haemolyticum***	**Coagulase-negative staphylococci**

^a^Species in bold indicate non-concordant results

^b^Species underlined are not included in the Accelerate identification panel

**Table 3:** Performance in species identification Accelerate PhenoTest™ BC kit in comparison to standard methods

	Identification (ID), *n* (%)			
	Correct	Correct genus^a^	False	Undetected/ unidentified^b^
Gram-negative rods				
v1.0 (*n* = 59/105, 56.2%)	54 (92%)			5 (8)^c^
SW1.2 (*n* = 27/61, 41%)	23 (92%)		2 (8)	
Staphylococci				
SW1.0 (*n* = 26/105, 24.8%)	23 (88)	1 (4)		2 (8)^c^
SW1.2 (*n* = 22/61, 36%)	20 (91)		2 (9)	
Streptococci				
SW1.0 (*n* = 11/105, 10.5%)	4 (36)	1 (9)	5 (45)	1 (9)
SW1.2 (*n* = 10/61, 16%)	4 (40)	3 (30)	2 (20)	1 (10)
Enterococci				
SW1.0 (*n* = 9/105, 8.6%)	7			2^c^
SW1.2 (*n* = 3/61, 5%)	3			

^a^For off-panel species

^b^For on-panel organisms

^c^Undetected Enterobacteriaceae (*n* = 3), staphylococci (*n* = 2), and enterococci (*n* = 2) were part of a polymicrobial sample

#### Polymicrobial samples

The PhenoTest™ BC kit is capable of identifying several different microorganisms within one sample. Monomicrobial results may be flagged “monomicrobial,” if the specific probe signal matches the universal signal in the FISH assay. Monomicrobial results may lack this comment, suggesting that the presence of further microorganisms cannot be ruled out.

Among the six polymicrobial samples investigated, one of two isolates (five samples) or one of three isolates (one sample) was detected using the PhenoTest™ BC kit (Table 2). All polymicrobial samples were identified by the initial Gram stain where possible and morphologically similar isolates were differentiated from the first subculture. Three of the six polymicrobial samples were flagged monomicrobial in the Accelerate run report. Overall, 66/92 (72%) monomicrobial and 3/6 polymicrobial samples were flagged monomicrobial (*κ* = 0.078, 95% confidence interval − 0.078 to 0.233).

#### Antimicrobial susceptibility testing

The PhenoTest™ BC kit comprises 9–14 antibiotics for Gram-negative rods, 6–7 antibiotics and 2 qualitative resistance phenotypes for staphylococci, and 5 antibiotics for enterococci. We chose to focus our evaluation on those antimicrobial drugs which are included in the panel routinely tested in our laboratory. Disk diffusion according to EUCAST guidelines served as reference. Susceptibility data for the isolates involved in this study are presented in Table 4. For Enterobacteriaceae, the performance of the PhenoTest™ BC kit was also compared to the performance of Vitek 2 because this system is frequently used in clinical microbiology laboratories.

**Table 4:** Susceptibility data for isolates using disk diffusion

Antimicrobial agent	SW1.0		SW1.2	
	Resistant, *n* (%)	Susceptible, *n* (%)	Resistant, *n* (%)	Susceptible, *n* (%)
Enterobacteriaceae				
Amikacin	0	49 (100)	0	19 (100)
Ertapenem	0	49 (100)	0	19 (100)
Ciprofloxacin	3 (6)	43 (94)	3 (16)	16 (84)
Ceftazidime	5 (10)	44 (90)	1 (5)	18 (95)
Gentamicin	1 (2)	48 (98)	2 (10)	17 (90)
Meropenem	0	49 (100)	0	19 (100)
Piperacillin-tazobactam	3 (6)	43 (94)	1 (5)	17 (90)
*Staphylococcus* species				
Cefoxitin	6 (30)	14 (70)	6 (30)	13 (70)
Erythromycin	5 (25)	15 (75)	6 (30)	13 (70)
Linezolid	0	20 (100)	0	19 (100)
Trimethoprim/sulfamethoxazole	4 (21)	15 (79)	0	11 (100)

##### Enterobacteriaceae

Among the 58 Gram-negative rods eligible for AST, 49/58 (89%) were correctly identified and AST results were obtained. Five isolates remained unidentified or undetected, and for four identified isolates, no AST could be provided. Results for seven antimicrobial agents were compared, including amikacin, ertapenem, ciprofloxacin, ceftazidime, gentamicin, meropenem, and piperacillin-tazobactam (Table 5). Overall, categorical agreement was reported for disk diffusion in 285/343 (83.1%) analyses, with 43/343 (12.5%) minor errors, 14/331 (4.2%) major errors, and 1/12 (8%) very major error. Ceftazidime showed poor agreement with the reference method. The majority of susceptible isolates (*n* = 44) were reported with intermediate (34/44, 77%) or resistant (10/44, 23%) MIC values. In addition, minor errors were recorded for 3/5 ceftazidime-resistant isolates. In comparison, Vitek 2 performed significantly better for ceftazidime (*P* < 0.0001). Errors, including a very major error, were also observed for piperacillin-tazobactam; however, the overall performance was comparable to Vitek 2.

**Table 5:** Antimicrobial susceptibility testing results for Enterobacteriaceae when compared to the reference method, disk diffusion

Antimicrobial agent		Categorical agreement^a^	Minor errors	Major errors	Very major errors
Amikacin					
A	Vitek 2	49 (100)	0	0	0
	SW1.0	49 (100)	0	0	0
B	Vitek 2	19 (100)	0	0	0
	SW1.2	19 (100)	0	0	0
Ertapenem					
A	Vitek 2	49 (100)	0	0	0
	SW1.0	49 (100)	0	0	0
B	Vitek 2	19 (100)	0	0	0
	SW1.2	19 (100)	0	0	0
Ciprofloxacin					
A	Vitek 2	49 (100)	0	0	0
	SW1.0	48 (98)	0	1 (2)	0
B	Vitek 2	18 (95)	1 (5)	0	0
	SW1.2	19 (100)	0	0	0
Ceftazidime					
A	Vitek 2	45 (92)^b^	3 (6)	0	1 (2)
	SW1.0	2 (4)	37 (76)	10 (20)	0
B	Vitek 2	19 (100)	0	0	0
	SW1.2	19 (100)^c^	0	0	0
Gentamicin					
A	Vitek 2	49 (100)	0	0	0
	SW1.0	47 (96)	2 (4)	0	0
B	Vitek 2	19 (100)	0	0	0
	SW1.2	19 (100)	0	0	0
Meropenem					
A	Vitek 2	49 (100)	0	0	0
	SW1.0	48 (98)	1 (2)	0	0
B	Vitek 2	19 (100)	0	0	0
	SW1.2	19 (100)	0	0	0
Piperacillin-tazobactam					
A	Vitek 2	46 (94)	2 (4)	0	1 (2)
	SW1.0	42 (86)	3 (6)	3 (6)	1 (2)
B	Vitek 2	19 (100)	0	0	0
	SW1.2	18 (95)	1 (5)	0	0

^a^In comparison to reference method (disk diffusion)

^b^SW1.0 versus Vitek 2, Fisher’s exact test, *P* < 0.0001

^c^SW1.0 versus SW1.2, Fisher’s exact test, *P* < 0.0001

##### Staphylococci

For staphylococci, results from the cefoxitin-screen, erythromycin and the macrolide-lincosamide-streptogramin B (MLSB) phenotype, and linezolid and trimethoprim/sulfamethoxazole testing were evaluated. Results were obtained for 20/23 (87%) eligible isolates, including a *Staphylococcus warneri* isolate identified as CoNS. For three *Staphylococcus aureus* isolates, no AST results were obtained. Agreement with disk diffusion was good with 92/100 (92%) concordant results (Table 6). For trimethoprim/sulfamethoxazole, 4/4 resistant CoNS isolates were reported as intermediate. The cefoxitin-screen failed for 2/20 (10%) samples. While these samples were reported monomicrobial by the PhenoTest™ BC kit, standard culture revealed the presence of a second staphylococcal isolate resistant to cefoxitin. Methicillin-resistant *S. aureus* (MRSA) were not isolated during the study period.

**Table 6:** Antimicrobial susceptibility testing results for staphylococci

	Categorical agreement^a^	Not analyzed	Minor errors	Major errors	Very major errors
Cefoxitin-screen					
SW1.0	18 (90)	2 (10)	–	0	0
SW1.2	17 (90)	0	–	1 (5)	1 (5)
Erythromycin					
SW1.0	19 (95)	1 (5)	0	0	0
SW1.2	19 (100)	0	0	0	0
MLSB					
SW1.0	20 (100)	0	–	0	0
SW1.2	18 (95)	0	–	0	1 (5)
Linezolid					
SW1.0	19 (95)	1 (5)	0	0	0
SW1.2	19 (100)	0	0	0	0
Trimethoprim/sulfamethoxazole					
SW1.0	16 (80)	0	4 (20)	0	0
SW1.2	11 (100)^b^	0	0	0	0

^a^In comparison to reference method (disk diffusion)

^b^Only for *Staphylococcus aureus*

##### Enterococci

*Enterococcus faecalis* and *Enterococcus faecium* were tested for ampicillin, linezolid, and vancomycin resistance. Results were obtained for 5/8 isolates. Two isolates from polymicrobial samples were not detected, and for one isolate, no AST results could be obtained. Complete categorical agreement was observed for the remaining five isolates.

### Evaluation of v1.2

To evaluate the performance of this commercially available version of the assay, 67 positive blood cultures were analyzed (Fig. 1b, Table 1).

#### Bacterial identification

Based on issues experienced with v1.0, software adjustments for microbial identification were made in v1.2. The species *S. warneri* and *Streptococcus oralis* were added to the group of detectable CoNS and *Streptococcus* species, respectively; *S. pneumoniae* was removed from the panel but instead reported on genus level. In addition, adjustments were made to minimize non-specific and indeterminate detections.

A total of 61 isolates were detected in the samples investigated during the second study period, 53/61 (87%) covered by the PhenoTest™ BC panel. By Gram stain and standard culture, 25 Gram-negative rods, 22 staphylococci, 10 streptococci, 3 enterococci as well as 1 Gram-positive rod were identified (Table 2, Table 3). The overall performance of v1.2 for bacterial identification was similar to v1.2 (Table 3). Consistent with findings using v1.0, differentiation among streptococci was poor. In addition, off-panel organisms (*Stenotrophomonas maltophilia*, *Haemophilus influenzae*, *Arcanobacterium haemolyticum*) were identified as CoNS, indicating poor specificity of this group-specific probe. In contrast to v1.0, false positive and “indeterminate” detections were no longer recorded (Table 2).

#### Polymicrobial samples

During the second study period, three polymicrobial samples with two (two samples) and three (one sample) isolates were identified by standard culture. The latter sample contained two different species of coagulase-negative staphylococci, which were counted as one isolate in this assessment. With this limitation, the sample was correctly and completely identified by the PhenoTest™ BC, however not eligible to AST because of morphological similarities of the included species, i.e., staphylococci and enterococci. In the remaining two samples, one of the isolates was missed or incorrectly identified (Table 2). Overall, 37/55 (67%) monomicrobial and 1/3 polymicrobial samples were flagged monomicrobial (*κ* = 0.092, 95% confidence interval − 0.082 to 0.267). In conclusion, the accuracy in detecting polymicrobial cultures was low and similar to that of SW1.0.

#### Antimicrobial susceptibility testing

##### Enterobacteriaceae

Among the 23 Gram-negative rods eligible for AST, 19/23 (83%) were correctly identified and AST results were obtained. The four remaining isolates were identified, but AST could not be provided. A major issue with SW1.0 was susceptibility testing for ceftazidime. The algorithm for this antimicrobial agent was adjusted in v1.2. Consequently, MIC values for sensitive isolates were correctly assigned (Table 5). For the remaining antimicrobial agents, the overall high degree categorical agreement with disk diffusion was confirmed.

##### Staphylococci

Results were obtained for 19/21 (90%) eligible isolates. Two *S. aureus* isolates were incorrectly identified as CoNS. For one of these isolates, AST results were presented but not considered for evaluation. Because of difficulties in detecting resistance to trimethoprim/sulfamethoxazole in CoNS using SW1.0, this antimicrobial combination was removed from the CoNS AST panel in SW1.2. With this adjustment, categorical agreement with disk diffusion was achieved for 84/87 (97%) analyses. In the cefoxitin-screen, 1/13 (8%) major and 1/6 very major error were noted for CoNS isolates. The inhibition zone diameters for these isolates were at the breakpoint, 25 and 24 mm, respectively. Therefore, they were also tested for the presence of the *mecA*-gene. In both cases, the molecular analysis confirmed the results obtained by disk diffusion. In addition to susceptibility to erythromycin, the PhenoTest™ BC kit includes an analysis to detect MLSB resistance. This phenotype was missed in 1/6 erythromycin-resistant isolates. This was the only isolate with an inducible resistance phenotype. Three of six isolates expressed constitutive clindamycin resistance, and 2/6 isolates displayed an efflux-mediated erythromycin resistance phenotype.

##### Enterococci

Results for the two enterococcal isolates eligible to AST in the second part of the study were in complete agreement with results from disk diffusion.

## Discussion

The rapid emergence of resistant bacteria is an obvious challenge for choosing early appropriate antibiotic treatment for severe infections including sepsis. Escalation and de-escalation of antibiotic treatment require information on antimicrobial susceptibility. In the present study, we evaluated the performance of the Accelerate Pheno™ system on clinical blood culture samples. The PhenoTest™ BC kit provided reliable identification and AST for major pathogens causing bloodstream infection, i.e., Enterobacteriaceae and *S. aureus*. Limitations were experienced in the detection of polymicrobial infections.

The PhenoTest™ BC kit panel covered 147/166 (88.6%) isolates included in the study. This coverage is very similar to a prospectively performed study on samples from pediatric oncology patients [7]. Among the on-panel microorganisms, 133/147 (90.5%) isolates could be detected and correctly identified by the Accelerate Pheno™ system. In addition, 5/19 (26%) off-panel microorganisms were detected and correctly reported as unidentified organisms. Thus, identification by the Accelerate Pheno™ system was overall comparable with other rapid methods such as the FilmArray Blood Culture ID (BCID) Panel, the Verigene Blood Culture tests for Gram-negative or Gram-positive bacteria, and MALDI-TOF MS directly from blood culture broth [1, 6, 11, 12, 15, 16, 22]. Highest performance was experienced for detection and identification of Enterobacteriaceae with 76/80 (95%) correct identifications, staphylococci with 42/46 (91%) correct identifications, and for enterococci with 10/11 (91%) correct identifications. Noticeable misidentifications occurred for *Streptococcus* species and off-panel organisms. This included cross-reactivity with off-panel species among the α-hemolytic streptococci, but also false identifications of streptococcal species as *S. pneumoniae* or CoNS, reducing also the positive predictive value for these staphylococcal species. Despite the clinical significance, differentiation of streptococcal species, and especially differentiation of *S. pneumoniae* from α-hemolytic streptococci, remains challenging [4, 21, 25]. Results from other rapid molecular identification methods are inconsistent [1, 6, 9, 19, 24]. Consequently, the species *S. pneumoniae* was removed from the species panel and this pathogen is reported on genus level by the PhenoTest™ BC kit software version v1.2. In addition, five off-panel organisms, including two β-hemolytic streptococci and two *S. aureus* isolates, were falsely identified as CoNS. Comparable data on β-hemolytic streptococci are not included in the previously published studies [7, 18]. Interestingly, and in contrast to the experience from the present study, CoNS were repeatedly reported as *S. aureus* in the study performed by Brazelton de Cardenas et al. [7]. Part of these misidentifications can be resolved by Gram stain. Based on our data, this complementation appears advisable and is also recommended by FDA.

The major achievement of the Accelerate Pheno™ system in comparison to rapid molecular tests is phenotypic AST for a panel of clinically relevant antimicrobial agents. Especially for Gram-negative bacteria and in regions with low prevalence of major resistance traits, phenotypic AST is still advisable for effective treatment choices but also for de-escalation of antimicrobial therapy [5, 17]. Overall, our results were similar to the studies by Marschal et al. [18] and Brazelton de Cardenas et al. [7]. Differences may be due to different reference methods and breakpoints, as well as differences in resistance patterns and species distribution among the investigated isolates. In addition to the previously published studies, we also investigated the performance of the PhenoTest™ BC kit operated with software version v1.2, which corresponds to the version available for clinical application. Ceftazidime was the antimicrobial agent most affected by the adjustments made in v1.2, and the results presented here are not reported in the previously published studies.

Overall, AST for Enterobacteriaceae was highly reliable. After adjustments made for ceftazidime in v1.2, categorical agreement of the PhenoTest™ BC-provided results with disk diffusion improved from 83.1 to 99.2%. A limitation of our study was the low number of resistant isolates investigated (Table 4). For ceftazidime, there was only one resistant isolate analyzed with v1.2. Five ceftazidime-resistant isolates identified during the first part of the study were therefore re-analyzed from simulated blood cultures (data not shown). Using SW1.2, 3/5 isolates were identified as resistant while 2/5 isolates were identified as susceptible in test results, suggesting that resistant strains including ESBL producers might be missed by the PhenoTest™ BC. 

Previous reports highlight difficulties in identifying resistance to cephalosporines and carbapenems [10, 13]. Therefore, in our laboratory and based on EUCAST recommendation, we complement Vitek 2 analyses with disk diffusion for piperacillin-tazobactam, ceftazidime and cefotaxime, as well as meropenem and ertapenem to avoid these system-related errors. The data presented here demonstrate that similar reservations might be necessary for interpretation of the PhenoTest™ BC. Only few ESBL-producing and no carbapenemase-producing Enterobacteriaceae were found among the isolates studied here. Hence, further studies are warranted to estimate the performance for these and other antibiotics to correctly identify resistant isolates.

For staphylococci, performance improved from 92% categorical agreement for SW1.0 to 97% for SW1.2. This was primarily due to the exclusion of trimethoprim/sulfamethoxazole from the CoNS AST panel. Since no trimethoprim/sulfamethoxazole-resistant *S. aureus* were isolated during this investigation, conclusions regarding the performance of the PhenoTest™ BC kit for trimethoprim/sulfamethoxazole are limited. Other errors occurred for qualitative analyses, i.e., cefoxitin-screen and MLSB detection. No MRSA occurred during the study period, but we tested 12 methicillin-resistant CoNS. Among these, 11 (92%) were identified correctly. Likewise, 8/8 constitutively clindamycin-resistant isolates were tested MLSB-positive; however, 1/1 inducibly resistant isolate was missed. The low frequency of these resistance phenotypes, and the lack of MRSA isolates, does not allow definitive conclusions and investigation of isolates across the entire range of MIC values is required to evaluate the performance of the PhenoTest™ BC kit.

## Conclusion

Our study demonstrates that the Accelerate Pheno™ system provides overall reliable identification results for Enterobacteriaceae and *S. aureus*. In combination with an initial Gram stain, the assay can therefore be used to complement routine methods for rapid preliminary identification and AST of major pathogens from bloodstream infections. Conclusions from this study are limited by the low frequency of resistant isolates investigated.

The current study focused on the analytical performance of the Accelerate Pheno™ system with promising results. However, the implementation of a rapid commercial AST system in the clinical routine is complex and depends also on the size of the laboratory, the cost of the system, cost per test, and the clinical performance of the method. Studies analyzing these factors are warranted prior to the implementation of the Accelerate Pheno™ system in other clinical diagnostic settings.
